# Four Unusual Cases of Congenital Forelimb Malformations in Dogs

**DOI:** 10.3390/ani11030813

**Published:** 2021-03-14

**Authors:** Simona Di Pietro, Giuseppe Santi Rapisarda, Luca Cicero, Vito Angileri, Simona Morabito, Giovanni Cassata, Francesco Macrì

**Affiliations:** 1 Department of Veterinary Sciences, University of Messina, Viale Palatucci, 98168 Messina, Italy; dipietros@unime.it (S.D.P.); francesco.macri@unime.it (F.M.); 2 Department of Veterinary Prevention, Provincial Health Authority of Catania, 95030 Gravina di Catania, Italy; grapisarda1975@gmail.com; 3 Institute Zooprofilattico Sperimentale of Sicily, Via G. Marinuzzi, 3, 90129 Palermo, Italy; giovanni.cassata@izssicilia.it; 4 Veterinary Practitioner, 91025 Marsala, Italy; vitoang@gmail.com; 5 Ospedale Veterinario I Portoni Rossi, Via Roma, 57/a, 40069 Zola Predosa (BO), Italy; morabitosimona759@gmail.com

**Keywords:** congenital limb deformity, dog, ectrodactily, syndactyly, ulnar hemimelia

## Abstract

**Simple Summary:**

Congenital limb defects are sporadically encountered in dogs during normal clinical practice. Literature concerning their diagnosis and management in canine species is poor. Sometimes, the diagnosis and description of congenital limb abnormalities are complicated by the concurrent presence of different malformations in the same limb and the lack of widely accepted classification schemes. In order to improve the knowledge about congenital limb anomalies in dogs, this report describes the clinical and radiographic findings in four dogs affected by unusual congenital forelimb defects, underlying also the importance of reviewing current terminology.

**Abstract:**

Four dogs were presented with thoracic limb deformity. After clinical and radiographic examinations, a diagnosis of congenital malformations was performed for each of them. In one case, a deformity involving both the radial and ulnar side of the distal limb was observed. Based on clinical and radiological evaluations, a diagnosis of postaxial terminal longitudinal ulnar hemimelia was performed. The term ectrodactyly was used to refer different malformations characterized by skin and soft tissue separation of the distal forelimb observed in two dogs. Simple complete uncomplicated syndactyly of the right forelimb, and complex incomplete uncomplicated syndactyly of the left forelimb were diagnosed in the fourth case. To the authors’ knowledge, ectrodactyly and simple complete uncomplicated syndactyly are very uncommon anomalies in companion animals and have been rarely documented. Moreover, postaxial terminal longitudinal ulnar hemimelia has still not been reported in dogs.

## 1. Introduction

Appendicular skeletal dysostoses are a group of dysmorphologies arising from alterations of the limb’s developmental process, resulting in a wide range of abnormalities involving individual bones or portions of bones of the growing embryo body extremities [1].

Hereditary (genetic aberrations) and environmental factors (drugs, maternal diseases, radiations and trauma) can cause an abnormal developmental process or an interference with a normal developmental process, respectively [2,3].

Limb formation is an intricate process, which takes place during the first weeks (from D23 to D35) of gestation in dogs [3,4,5] Presumptive limb-forming fields are early established during embryonic development because of the rounding up of the somatic mesoderm cells of the hypomere beneath the surface ectoderm [5,6].

Congenital defects and varying associations of stilopodium, zeugopodium and autopodium dysmorphologies have been previously described in cats and dogs. [7,8,9,10,11,12]. The term stylopodium refers to a limb that terminates anywhere along the femur or humerus; zeugopodium is a limb that terminates along the radius and ulna or tibia and fibula, and autopodium when a limb terminates at the bones of the manus [13].

Recent reports of forelimb and hindlimb malformations of carnivores include ectrodactyly [14], preaxial longitudinal intercalary radial hemimelia [15], simple syndactyly and unilateral pelvic limb adactyly in puppies [16,17], absence of the humerus with preaxial terminal longitudinal hemimelia and hypoplasia of the scapula in a dog [17] and partial paraxial radial hemimelia [18].

Despite the growing number of reports, in dogs the current knowledge about congenital limb deformity is very poor. Moreover, compared to human literature, in veterinary medicine a complete and unequivocal description of many congenital defects is still lacking.

In order to improve the knowledge about congenital limb anomalies in dogs, the aim of this report is to describe the clinical and radiographic findings in four dogs affected by dysostoses of the distal extremities.

The importance of this report lies in it helping the clinician to recognize more easily the features of rarely reported congenital limb deformities in canine species in order to perform a diagnosis.

## 2. Case Presentations

### 2.1. Ethical Statement

No ethical approval was required in compliance with European Directive 2010/63/UE and Italian Regulation D.Lgs n. 26/2014 because all the data derived from routine veterinary clinical practices.

### 2.2. Clinical Examination

Four client-owned, crossbreed young dogs were presented each with an obvious deformity in the forelimbs. All dogs had an unknown history and the anomalies of forelimbs were supposed to be congenital. The sites of deformation were assessed by physical and radiographic examination. Forelimb radiographs were obtained using an Analogic Radiographic/Fluoroscopic Table System (Dedalus Mb 90/20 IMX-2A, Imago Radiology S.r.l., 20081, Abbiategrasso (MI), Italy) with a digital radiography system (Fujifilm Medical Systems, Cernusco sul Naviglio (MI), Italy) in different projections: mediolateral, oblique and dorsopalmar views. The following X-ray exposure setting was used: 40–60 kV, 8–12.5 mAs, 100 cm film-focus distance, no grid.

Below, the physical and radiographic findings of enrolled dogs are described.

### 2.3. Case 1

A two-year-old male dog was referred for a nonfunctional right forelimb due to decreased range of motion of the right elbow joint and moderate disuse muscle atrophy with no pain during palpation.

**Figure 1:**
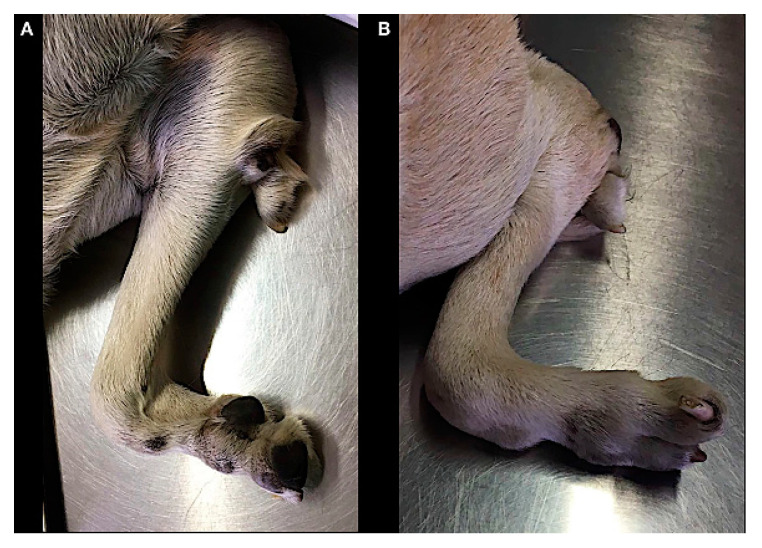
Case 1. Medial (**A**) and lateral (**B**) views of the right forelimb.

On physical examination, coexistent clinical abnormalities were a reduced number of digits (hypodactlyly) with a complete rotation of the lateral nail, and an ectopic digit-like structure in the elbow region. Three complete footpads (metacarpal pad and two digital pads) were observed. A footpad was also present in the digit like structure (Figure 1).

**Figure 2:**
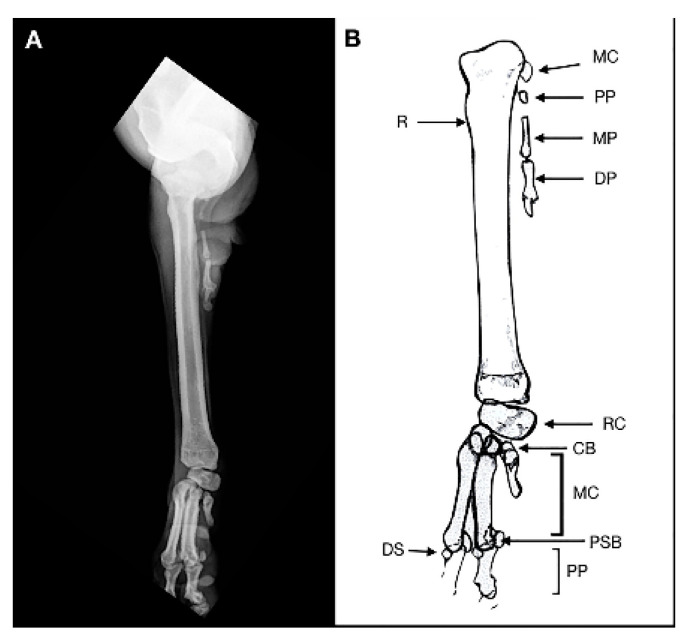
Case 1. Craniocaudal radiograph (**A**) and drawing scheme (**B**) of the right forelimb malformation. R: Radius; RC: radial carpal bone; CB: carpal bone; MC: metacarpal bone; PP: proximal phalange; MP: middle phalange; DP: distal phalange; DS: dorsal sesamoid; PSB: proximal sesamoid bone.

**Figure 3:**
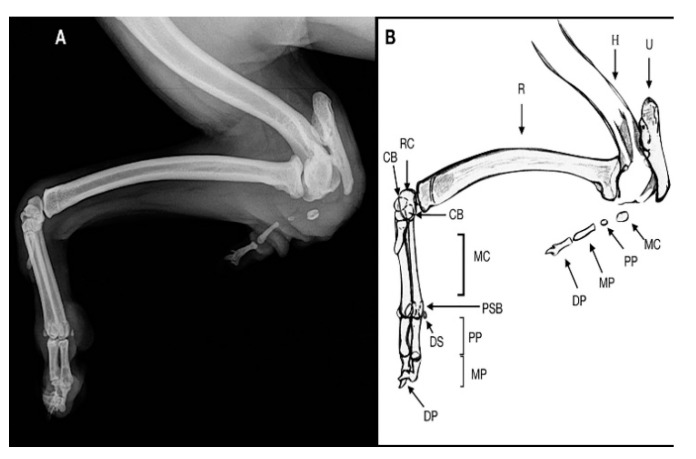
Case 1. Mediolateral radiograph (**A**) and drawing scheme (**B**) of the right forelimb malformation. H: humerus; U: ulna; R: radius; RC: radial carpal bone; CB: second and third distal carpal bone; MC: metacarpal bone; PP: proximal phalange; MP: middle phalange; DP: distal phalange; DS: dorsal sesamoid; PSB: proximal sesamoid bone. The olecranon and trochlear notch were hypoplastic.

The radiographic examination consisted of two views, including a mediolateral view of the forelimb section and a dorsoplantar view of the manus (Figure 2 and Figure 3).

In the mediolateral view of the forelimb section, a severe deformity was observed due to a collapse of the elbow joint, which was in acute flexion. The radial head and the humeral condyle were deformed, showing a rounded and irregular articular surface. The ulna showed only the olecranon, whereas the medial and distal ulnar portions were missing.

The olecranon and trochlear notch were hypoplastic. This may suggest that the trochlear notch dislocated long before presentation, explaining the stiffness of the elbow joint.

In the soft tissue near the caudal surface of the radial head a rudimentary digit containing a hypoplastic metacarpal bone and proximal, middle and distal phalanx, without evidence of joints, was observed, suggestive of an ectopic digit-like structure. It was characterized by the outline of the hypoplasic fifth metacarpal bone and the proximal, medial and distal phalanges of the fifth digit. The dorsopalmar view of the manus showed no ulnar carpal bone, accessory bone and distal line of carpal bones. The radial carpal bone, two complete digits and a hypoplasic metacarpal bone without its phalanges, were noted. A 180° rotation on the axial plane of the lateral distal phalange was observed. The remaining digits and soft tissue were within the normal limits. Clinical and radiographic findings showed a postaxial terminal longitudinal ulnar hemimelia with absence mid and distal ulna, ulnar and accessory carpal bone, distal carpal bone IV and an ectopic fifth digital ray.

No treatment was performed for managing the congenital deformity because the dog had no evidence of discomfort.

### 2.4. Case 2

**Figure 4:**
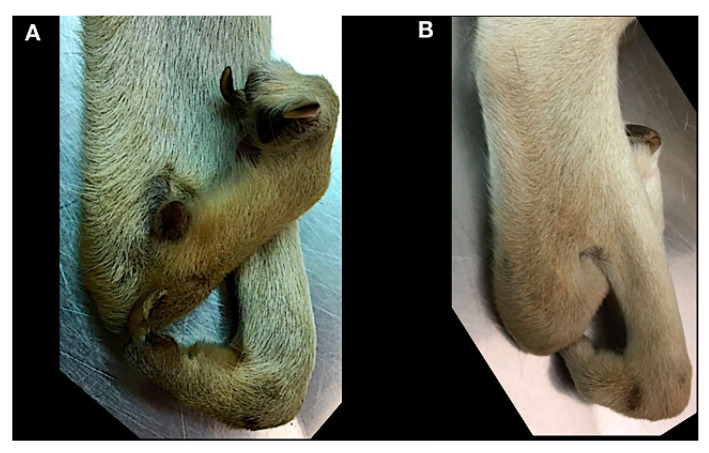
Case 2. Medial (**A**) and lateral (**B**) views of the right forelimb.

An 11-month-old female dog was referred for a nonfunctional right forelimb without pain at palpation. The clinical examination showed a lobster’s claw involving the paw, carpus and antebrachium. The skin and soft tissues of the distal limb were split between radius and ulna up to the proximal third of the antebrachium. The ulnar carpal bone, metacarpal bone and phalanges showed a 180° rotation on the axial plane. The ulnar carpal joint was in acute cranial flexion, with a mediocaudal rotation of the manus moving medial to the radio. The radial carpal joint was also in acute flexion (Figure 4).

**Figure 5:**
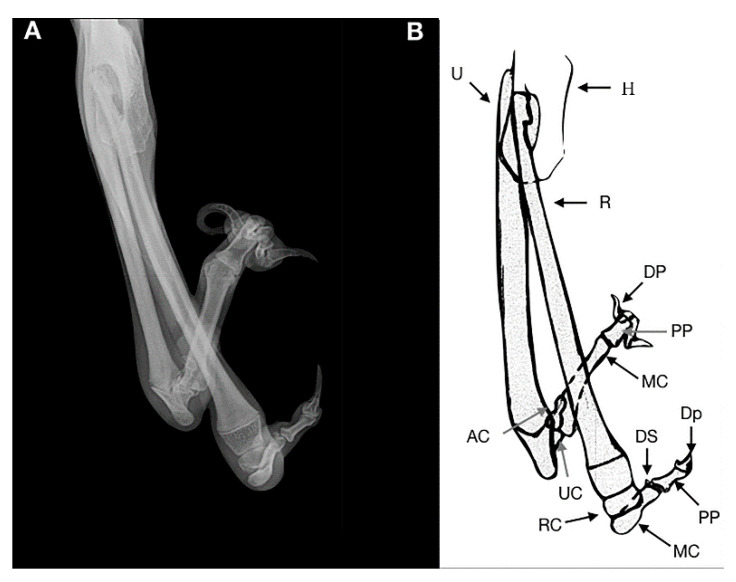
Case 2. Craniocaudal radiograph (**A**) and drawing scheme (**B**) of the right forelimb malformation. R: Radius; U: Ulna; H: Humerus; RC: Radial Carpal Bone; UC: Ulnar Carpal Bone; AC: Accessory Bone; MC: Metacarpal Bone; PP: Proximal Phalange; DP: Distal Phalange; DS: Dorsal Sesamoid. Note the “stubby bone” (arrows) resulting by the fusion of the two proximal phalanges.

**Figure 6:**
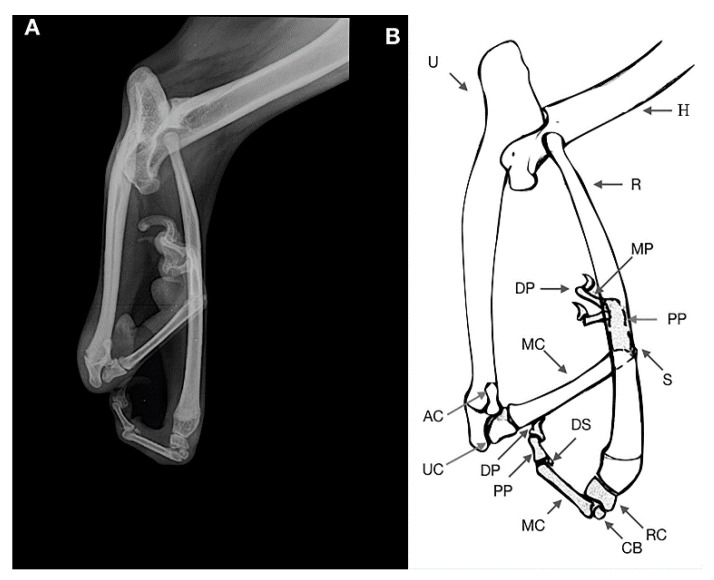
Case 2. Mediolateral radiograph (**A**) and drawing scheme (**B**) of the right forelimb malformation. R: Radius; U: Ulna; H: Humerus; RC: Radial Carpal Bone; UC: Ulnar Carpal Bone; AC: Accessory Bone; CB: Carpal Bone; MC: Metacarpal Bone; PP: Proximal Phalange; MP: Middle Phalange; DP: Distal Phalange; DS: Dorsal Sesamoid. Arrows indicate the “stubby bone” resulting by the fusion of the two proximal phalanges.

The radiographic examination consisted of mediolateral and dorsocaudal views of the right forelimb section (Figure 5 and Figure 6), where a malformation of the elbow with proximal dorsal radial luxation was observed.

A severe hypoplasia of the proximal epiphysis of the radius was observed, the radial head showed a severe deformation with the flattening of both medial and lateral epicondyles and an increased dimension of the radial fossa was noted. The olecranon was lengthened and flattened.

A soft tissue separation between radius and ulna along the interosseous ligament and a severe increase of the intraosseus distance were observed. The ulna was shorter than radius. The radius-carpal joint was normal with a short bone compatible with the radial carpal bone, which in turn was articulated with a carpal bone of the second line. The carpal joint was in acute flexion. One metacarpal bone, dorsal and ventral sesamoids and two phalanges of the first digits (proximal and distal phalanges) were noted.

The distal epiphysis of the ulna was articulated with the ulnar carpal bone and accessory bone. This joint was in acute cranial flexion showing a 180° rotation on the axial plane, with a mediocaudal rotation of the manus moving medial to the radio. One metacarpal bone and one dorsal sesamoid were noticed; we assumed a fusion of the two proximal phalanges resulting in one “stubby bone”. Two medial and distal phalanges were articulated with the stubby bone. Clinical and radiographic findings were consistent with ectrodactyly.

No treatment was considered at time for managing the congenital deformity.

### 2.5. Case 3

**Figure 7:**
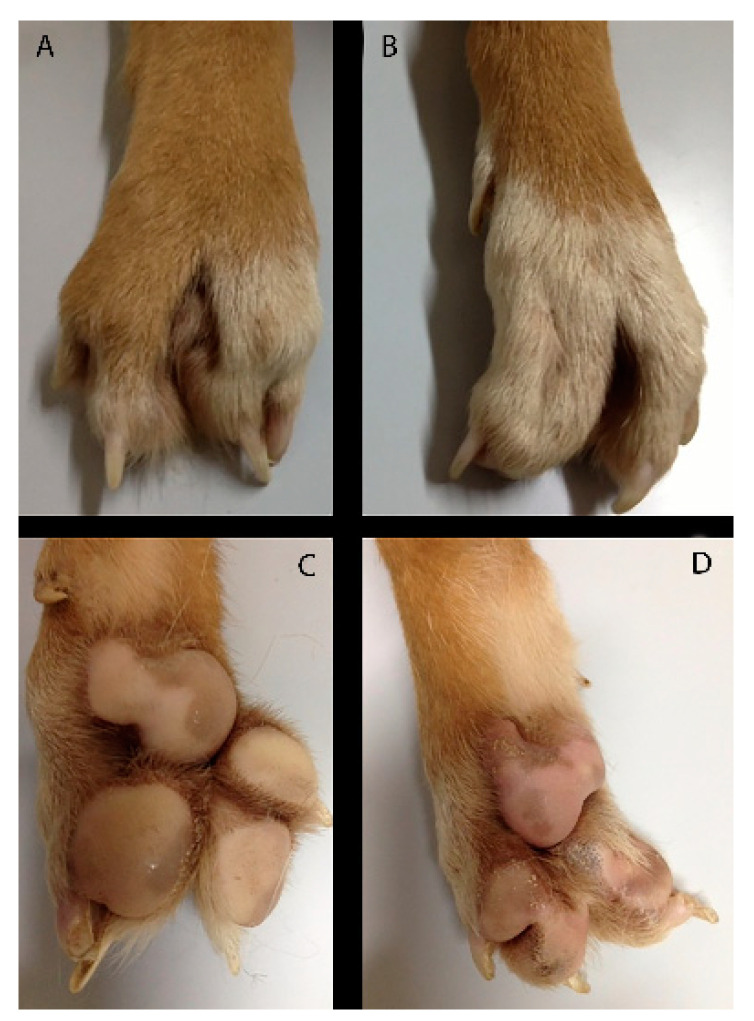
Case 3. Dorsal and Palmar views of both forelimbs. Right manus (**A**,**C**) and left manus (**B**,**D**).

**Figure 8:**
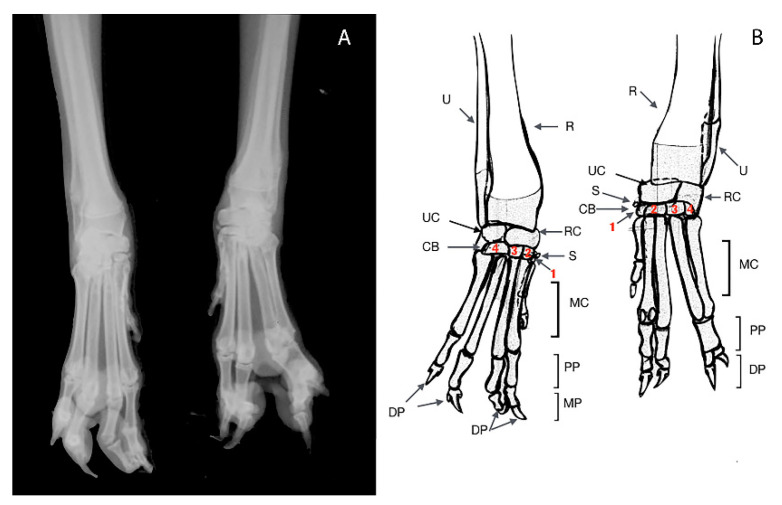
Case 3. Dorsopalmar radiograph (**A**) and drawing scheme (**B**) of the both forelimbs malformation. R: Radius; U: Ulna; RC: Radial Carpal Bone; UC: Ulnar Carpal Bone; CB: Carpal Bone; 1–4: Distal Carpal Bones; MC: Metacarpal Bone; PP: Proximal Phalange; MP: Middle Phalange; DP: Distal Phalange; DS: Dorsal Sesamoid; S: Sesamoid Bone.

A 10 months-old male dog was referred for an abnormal aspect of both front paws, not associated with lameness. Physical examination of the involved limb revealed a deformity of the paw characterized by a cutaneous absence of normal separation between II–III digits and IV–V digits. The right paw showed one metacarpal pad and three digital pads, whereas the left paw showed one metacarpal pad and two digital pads (Figure 7). The radiographic examination consisted of dorsopalmar view of the manus (Figure 8).

In the left manus, a complete bony fusion between proximal phalanges of the fourth and fifth digit was noted.

Clinical and radiographic findings were a simple complete uncomplicated syndactyly of the right limb and a complex incomplete uncomplicated syndactyly of the left limb.

The dog had no evidence of discomfort, so that no treatment was performed.

### 2.6. Case 4

**Figure 9:**
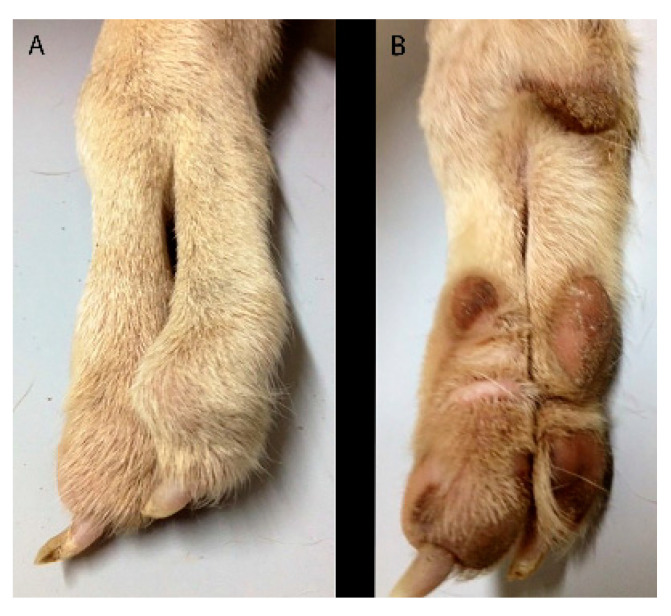
Case 4. Dorsal (**A**) and palmar (**B**) views of the right forelimb. Note the deformity of the paw with only two digits and a “cleft hand aspect”.

**Figure 10:**
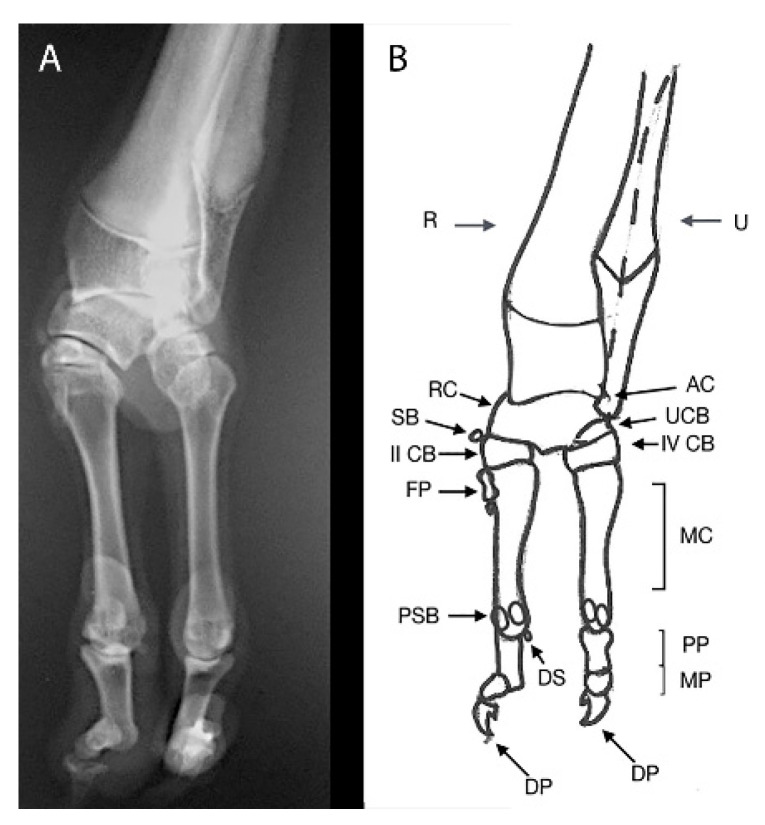
Case 4. Dorsopalmar radiograph (**A**) and drawing scheme (**B**) of the right forelimbs malformation. R: Radius; U: Ulna; RC: Radial Carpal Bone; UCB: ulnar Carpal Bone; AC: Accessory Bone; SB: Sesamoid Bone; CB: Carpal Bone; MC: Metacarpal Bone; PP: Proximal Phalange; MP: Middle Phalange; DP: Distal Phalange; DS: Dorsal sesamoid; PSB: Proximal Sesamoid Bone.

A one-year-old male dog was referred for right forelimb lameness. Physical examination revealed a deformity of the paw characterized by the presence of only two digits and a “cleft hand aspect”. Two digital pads in each paw and one carpal pad in the palmar view were observed (Figure 9). The radiographic examination consisted of the dorsopalmar view of the right manus. (Figure 10).

On X-ray, normal radius and ulna bones, deformed radial and ulnar carpal bones, small and deformed carpal bones and the presence of only two complete digits articulated with carpal bones of the distal line, were noticed. A digit showed one supernumerary footpad, instead the other finger a hypoplasic footpad. Clinical and radiographic findings were consistent with ectrodactyly. No treatment was performed for managing the bone deformity.

## 3. Discussion

In veterinary medicine, Nomina Embryologica Veterinaria (NEV) (2017) is the only classification system recognized that allows classifying many congenital limb malformations. However, it needs to be expanded in order to improve and facilitate communication about the specific characteristics of congenital anomalies in animals [3,19,20,21]. It only lists malformations, without defining and describing them, and some deformities are listed more than once under different names. The NEV is not canine specific. This is one reason why manuscripts such as the current work are important.

In this study we reported the clinical and radiological patterns of four appendicular skeletal dysostosis in canine species. Clinical observation is only the first step in the diagnosis of bone malformations, so that radiographic evaluations are essential to evaluate the extent of deficits.

In case 1, a malformation involving the antebrachium and carpal bones and paw of the right thoracic limb was reported. An additional feature was the presence of a floating digit-like structure without any bony attachment to the elbow joint. This congenital defect was identify as postaxial terminal longitudinal ulnar hemimelia.

To the authors’ best knowledge, no similar deformities have been reported in dogs, other animal species or humans, although a variable degree of ulna and digit deficiency has been observed in the latter [22,23]. This can make description difficult. The dog showed unilateral ulnar defect associated with other congenital alterations involving the carpus, both medial and lateral digital rays and the elbow joint. It was consistent with previous reports of human ulnar longitudinal deficiency [21,24].

In case n.2 and 4, a diagnosis of ectrodactyly was performed, according to clinical cases of limb malformations previously described [9,25]. In both cases, radiography permitted detection of the absence or hypoplasia of phalangeal bones, metacarpal bones, carpal bones and the hard tissues fusion or separation [26,27,28,29]. Splitting of the skin and soft tissues between radius and ulna to the level of the proximal third of the antebrachium was the prominent feature in case 2. The absence of three digits associated with a deep, v-shaped, excessive interdigital space between the remainders was the main finding in case 4.

In case 3, a diagnosis of syndactyly of the pectoral limbs was performed. To our knowledge, no report of simple complete uncomplicated syndactyly of the right forelimb and complex incomplete uncomplicated syndactyly of the left forelimb exists, as described in this report.

Over time, the term “hemimelia” has been adopted for better specifying congenital deficits of limbs, where one or more bones were totally or partially missing [2,17].

In veterinary medicine, different patterns of this malformation were classified as hemimelia terminal, when all or parts of the middle and distal bones were absent and hemimelia intercalary when segments of the middle portion of the limb were lacking, while the located proximally and distally structures developed normally [30]. Each of these latter varieties, in turn, is also qualified as transverse or longitudinal, depending on whether bones are missing across the limb’s width or along the preaxial/postaxial side of the limb [31].

Canine defects of the medial and central rays of the limb bud have been previously described and named as radial hemimelia and ectrodactyly [14,17,26]. Moreover, both ulnar ray abnormalities confined to the paw and ulnar ray defects as partial symptoms of other malformations have been also reported in canine species [9,26,32].

In canine medicine, the term hemimelia has previously proved useful to identify which segments of the limb were misshapen or missing [17]. However, the term postaxial terminal longitudinal ulnar hemimelia is not sufficient to provide a complete description of the range of abnormalities occurred in the paw, forearm and elbow in the present case. When describing the present dysmorphology, particular attention should be taken in reporting not only ulnar-side defects, but also the absence of metacarpal bone and phalanges of the first digit.

In humans, some studies permitted detection and classification of complex forms of ulnar longitudinal deficiencies, such as those characterized by the presence of radial-side abnormalities of the manus [33]. Moreover, amongst the manus abnormalities, thumb and first web deficiencies are noted frequently in congenital defects of bony and soft tissue elements of the forearm ulnar border. In these cases, thumb defects are recognized as a surgical priority because of the importance of restoring the opposition function [33]. In dogs, the first digit is not part of the weightbearing surface of the limb and does not exert the function of grasping items. Dogs bear weight on digits II to V, with main weight bearing occurring on digits III and IV, so that it may be possible to bear body weight without the fourth digit [34].

The term “ectrodactyly” means one or more digits are missing or not fully developed, even though in veterinary medicine this term was also used to indicate a median distal cleft of the limb [9,12,14,35]. This condition is very heterogeneous, due to the different morphological features. Clinical diversity occurs not only between animals, but also between legs of the same subject. The deformity is usually unilateral and may be associated with soft tissue contractures, limited range of motion in the carpus and elbow luxation [26,36]; it is also associated with concurrent vertebral malformation [9].

Some authors reported that soft tissue separation involves the metacarpus, whilst distal cleft of the limb extended up the carpus joint occurs sporadically in dogs [36]. Few cases of complete parasagittal plane separation of the antebrachium between medial and lateral segments have been previously described [26,37]. Surgical therapy should be performed to relieve pain, guaranteeing aesthetic and functional recovery and preventing progressive limb deformities [14,26,38].

For this kind of malformation, the use of the generic term ectrodactyly does not aid in planning of treatment because it is used to identify different anomalies, such as oligodactyly and cleft of the foot, paw or limb [14]. Schistomelia is another term listed in Nomina Embryologica Veterinaria [20] that refers to the split of the limb. However, this definition has never been mentioned in previous reports. Other terms such as brachydactyly and aphalangia have been used to describe malformations of the distal limb in veterinary medicine [3,10,17].

In case 3, the diagnosis of syndactyly of the pectoral limbs was based on the human nomenclature, already widely accepted in veterinary medicine. A simple syndactyly occurs when adjacent digits are merged only by skin and soft tissue. A complex syndactyly is characterized by the lack of separation of hard tissues, in addition to skin and soft tissue abnormalities. Syndactyly may further be qualified as complete, when digits are interconnected throughout their entire length, and uncomplete when total connection of adjacent digits does not occur. If other anomalies are associated with syndactyly, the term complicated syndactyly may be adopted, whereas the definition of uncomplicated syndactyly should be used otherwise [39,40,41]. Previous reports treated several patterns of this congenital defect in dog [3,17,35,39,42,43].

Although little is known about the basic mechanisms of congenital limb deformities, several aetiological factors have been identified [44]. Causes of very similar anomalies to those described in the present case reports include genetic defects, administration of chemotherapeutics, malnutrition (lack of riboflavin), intake of drugs such as thalidomide or corticosteroids (in chick embryos), transplacental virus infections and X-rays [45,46,47]. Other causes of distal limb absence in young animals include strangulation by restrictive bands, in utero accidents and postnatal trauma [48].

In our cases, there was no information concerning traumatic events, inbreeding and/or environmental or genetic factors, so that the aetiology of these abnormalities remains unknown.

## 4. Conclusions

This report improves the literature about congenital limb deformities, describing the patterns of rarely reported lesions and discussing how difficult it is to know the correct identification and classification.

A well-defined classification scheme of congenital dysmorphologies of the forelimb is advisable. Novel nomenclature should take into consideration both the genetic basis and the wide spectrum of morphological features of limb dysostosis.

Extrapolation from the human literature has been considered of limited value [14] but in our opinion it may serve some important cues.

Likely, the real prevalence of these conditions in companion animals is little known; therefore, further reports are required.

## Data Availability

The data presented in this study are available on request from the corresponding author.

## References

[1] Breur G.J., Lust G., Todhunter R.J. (2009). Genetics of canine hip dysplasia and other orthopaedic traits. The Genetics of the Dog.

[2] Towle H.A.M., Breur G.J. (2004). Dysostoses of the canine and feline appendicular skeleton. J. Am. Vet. Med. Assoc..

[3] Di Dona F., Della Valle G., Meomartino L., Lamagna F., Fatone G. (2016). Congenital deformity of the distal extremities in three dogs. Open Vet. J..

[4] Ruvinsky A., Ruvinsky A., Sampson J. (2001). Developmental Genetics. The Genetics of the Dog.

[5] Gilbert S.F. (2003). Development of the tetrapod limb. Developmental Biology.

[6] Noden D., De Lahunta A., Noden D.M., De Lahunta A. (1985). Limb development In The Embryology of Domestic Animals: Developmental Mechanisms and Malformations.

[7] Blevins W.E., Breur G.J., Kim J. (2006). Morphological and functional evaluation of a dog with dimelia. Vet. Comp. Orthop. Traumatol..

[8] Montgomery R., McEwen V., Lockwood A. (2009). Bilateral radial hemimelia, polydactyly and cardiomegaly in two cats. Vet. Comp. Orthop. Traumatol..

[9] Carvallo F.R., Domínguez A.S., Morales P.C. (2011). Bilateral ectrodactyly and spinal deformation in a mixed-breed dog. Can. Vet. J..

[10] Macrì F., Lanteri G., Rapisarda G., Marino F. (2012). Unilateral forelimb partial aphalangia in a kitten. J. Feline Med. Surg..

[11] Pisoni L., Cinti F., Del Magno S., Joechler M. (2012). Bilateral radial hemimelia and multiple malformations in a kitten. J. Feline Med. Surg..

[12] Wrzosek M., Płonek M., Zeira O., Bieżyński J., Kinda W., Guziński M. (2014). Congenital bipartite atlas with hypodactyly in a dog: Clinical, radiographic and CT findings. J. Small Anim. Pr..

[13] Macrì F., De Majo M., Rapisarda G., Mazzullo G. (2009). Two cases of feline ectromelia: Autopodium ectromelia associated with humero-ulnar synostosis and zeugopodium ectromelia. J. Feline Med. Surg..

[14] Barrand K.R. (2004). Ectrodactyly in a West Highland white terrier. J. Small Anim. Pract..

[15] Alam M.R., Heo S.Y., Lee H.B., Kim J.H., Park Y.J., Lee K.C., Choi I.H., Kim N.S. (2006). Preaxial longitudinal intercalary radial hemimelia in a dog: A case report. Vet. Med..

[16] Macrì F., Marino F., Rapisarda G., Lanteri G., Mazzullo G. (2010). A Case of Unilateral Pelvic Limb Adactyly in a Puppy Dog. Anat. Histol. Embryol..

[17] Macrì F., Ciotola F., Rapisarda G., Lanteri G., Albarella S., Aiudi G., Liotta L., Marino F. (2013). A rare case of simple syndactyly in a puppy. J. Small Anim. Pr..

[18] Macrì F., Di Pietro S., Palumbo Piccionello A., Rapisarda G., Lanteri G., Angileri V., Marino F. (2017). A rare case of partial paraxial radial hemimelia in a puppy: A case report. Vet. Med..

[19] Cornillie P., Van Lancker S., Simoens P. (2004). Two Cases of Brachymelia in Cats. Anat. Histol. Embryol..

[20] World Association of Veterinary Anatomists (2017). Nomina Embryologica Veterinaria (Second Edition). http://www.wavaamav.org/Downloads/nev_2017.pdf.

[21] Ogino T. (2007). Clinical features and teratogenic mechanisms of congenital absence of digits. Dev. Growth Differ..

[22] Kornak U., Mundlos S. (2003). Genetic Disorders of the Skeleton: A Developmental Approach. Am. J. Hum. Genet..

[23] Al-Qattan M., Al-Sahabi A., Al-Arfaj N. (2010). Ulnar Ray Deficiency: A Review of the Classification Systems, the Clinical Features in 72 Cases, and Related Developmental Biology. J. Hand Surg..

[24] Afzal M., Malik S. (2014). Longitudinal deficiency of upper limb: Similar case presentation of two subjects with unilateral ulnar hemimelia, carpal and metacarpal deficiency, and severe oligodactyly. Asian Biomed..

[25] Lee M.-I., Kwak H.-H., Kim J.-H., Shin H.-S., Woo H.-M., Kang B.-J. (2020). Surgical Ectrodactyly Repair Using Limb-lengthening and Bone Tissue Engineering Techniques in a Toy Dog Breed. In Vivo.

[26] McKee W.M., Mitchell R.A.S., Innes J.F., Lascelles B.D.X., Johnson K.A. (2001). Surgical reconstruction of ectrodactyly deformity in four dogs. Vet. Comp. Orthop. Traumatol..

[27] Ferreira M.P., Alievi M.M., Dal-Bó I.D.S., Nóbrega F.S., Gonzalez P.C.S., Beck C.A.D.C. (2016). Surgical management of ectrodactyly in a dog. Semina Ciências Agrárias.

[28] Tchaprazov T., Kostov D., Vladova D. (2007). A case of ectrodactyly in a chow chow dog. Trakia. J. Sci..

[29] Harasen G. (2010). Surgical management of ectrodactyly in a Siberian husky. Can. Vet. J. La Rev. Vet. Can..

[30] Frantz C.H., O’Rahilly R. (1971). Ulnar hemimelia. Artif. Limbs.

[31] Towle H.A., Breur G.J., Tobias K.M., Johnston S.A. (2012). Miscellaneous orthopedic conditions. Veterinary Surgery: Small Animal.

[32] Alonso R.A., Hernandez A., Diaz P., Cantú J.M. (1982). An autosomal recessive form of hemimelia in dogs. Vet. Rec..

[33] Cole R.J., Manske P.R. (1997). Classification of ulnar deficiency according to the thumb and first web. J. Hand Surg..

[34] Riegger-Krugh C., Millis D.L., Weigel J.P. (2014). Canine Anatomy. Canine Rehabilitation and Physical Therapy.

[35] Pratschke K. (1996). A case of ectrodactyly in a dog. Irish. Vet. J..

[36] Carrig C., Wortman J., Morris E., Blevins W., Root C., Hanlon G., Suter P. (1981). Ectrodactyly (split‐hand deformity) in the dog. Vet. Radiol..

[37] Mehrjerdi H.K., Hayati F., Sardari K., Mirshahi A., Gachpaz S. (2008). Ectrodactyly in a mix breed dog. Iran. J. Vet. Surg..

[38] Pisoni L., Del Magno S., Cinti F., Dalpozzo B., Bellei E., Cloriti E., Joechler M. (2014). Surgical induction of metacarpal synostosis for treatment of ectrodactyly in a dog. Vet. Comp. Orthop. Traumatol..

[39] Towle H., Friedlander K., Ko R., Aper R., Breur G. (2007). Surgical treatment of simple syndactylism with secondary deep digital flexor tendon contracture in a Basset Hound. Vet. Comp. Orthop. Traumatol..

[40] Kozin H.S. (2001). Syndactyly. J. Hand Surg..

[41] Dao K.D., Shin A.Y., Billings A., Oberg K.C., Wood V.E. (2004). Surgical Treatment of Congenital Syndactyly of the Hand. J. Am. Acad. Orthop. Surg..

[42] Richardson E.F., Wey P.D., Hoffman L. (1994). Surgical management of syndactyly in a dog. J. Am. Vet. Med. Assoc..

[43] Schultz V., Watson A.G. (1995). Lumbosacral transitional vertebra and thoracic limb malformations in a Chihuahua puppy. J. Am. Anim. Hosp. Assoc..

[44] Gilbert S.F. (2000). Development Biology.

[45] Johnson E.M., Wilson J.G., Warkany J. (1965). Nutritional factors in mammalian teratology. Teratology, Principles and Techniques.

[46] Karnofsky D.A., Wilson J.G., Warkany J. (1965). Mechanism of action of certain grow—thinhibiting drugs. Teratology, Principles and Techniques.

[47] Warkany J., Wilson J.G., Warkany J. (1965). Development of experimental mammalian teratology. Teratology, Principles and Techniques.

[48] Johnson K.A., Watson A.D.J., Page R.L., Ettinger S.J., Feldman E.C. (1995). Skeletal diseases. Textbook of Veterinary Internal Medicine.

